# The effect of activated silicon dioxide and betaine supplementation on quails’ growth and productivity

**DOI:** 10.14202/vetworld.2021.2009-2015

**Published:** 2021-08-05

**Authors:** Adi Ratriyanto, Sigit Prastowo, Nuzul Widyas

**Affiliations:** Department of Animal Science, Faculty of Agriculture, Universitas Sebelas Maret, Jl. Ir. Sutami 36A, Surakarta 57126, Indonesia

**Keywords:** betaine, follicle, productive parameters, quail performance, silicon dioxide

## Abstract

**Background and Aim:**

Silicon dioxide and betaine supplements are essential in the poultry diet to improve growth and laying performance. This study aimed to determine the effect of activated silicon dioxide (ASD) and betaine supplementation on quails’ growth performance during the starter period and on follicular development and productive parameters at the onset of the laying period.

**Materials and Methods:**

The research used 1064 quails, aged 7 days. Four treatments were employed: A basal diet (control), a diet supplemented with 200 ppm ASD, a diet supplemented with 0.12% betaine, and a diet supplemented with a combination of 200 ppm ASD and 0.12% betaine (ASD+Betaine). Each diet group included seven replicates of 38 quails. The observed parameters were the quails’ growth performance during the starter period and follicular development and egg production during the onset of the laying period.

**Results:**

The results showed that combined supplementation with ASD+Betaine improved the quails’ growth performance during the starter period (p<0.05). However, these supplements, either as single feed additives or in combination, did not affect ovarian and follicular characteristics during the onset of the laying period. Still, ASD and betaine enhanced egg production and weight. The highest magnitude of change resulted from combined supplementation with ASD+Betaine (p<0.05).

**Conclusion:**

The ASD+Betaine could be productively applied to quails’ diets during the starter period and at the onset of the laying period.

## Introduction

Quails are potential egg producers and have been widely and intensively farmed [1]. Their small size and weight give them the potential to increase farmers’ incomes because it costs less for farmers to maintain them [2]. Female quails (in this case, *Coturnix coturnix japonica*) reach sexual maturity at approximately 6-7 weeks of age, with a body weight of around 110-117 g [3,4], and they get to the peak of their egg production at the age of 15 weeks [5,6]. Egg production patterns are genetically programmed, but they may be modified by environmental factors, such as nutrition [7,8]. Kaye *et al*. [1] suggested that a high metabolic rate causes high egg production in quails, and nutritional modifications, such as adding supplements to feed, are commonly made to increase egg production [9,10].

Activated silicon dioxide (ASD) is a natural mineral derived when pure silicon dioxide is activated by subjecting micronized silicon dioxide powder to an electromagnetic treatment [11]. Biologically, it applies a specific quantum of electromagnetic energy inside the gut, increasing the electronegativity of water and, thus, accelerating ion exchange [11,12]. In contrast to other silicon dioxide compounds, ASD has an organized structure, greater energy transfer potential, and greater surface contact with the environment [13,14]. ASD accelerates biochemical changes in the digestive tract, including enzymatic digestion, nutrient ionization, and nutrient transfer across cells. The cation exchange and absorption properties of silica, such as increasing feed retention time and decreasing intestinal emptying rates, improve nutrient absorption and feed efficiency in poultry [15-17]. ASD supplementation improves growth performance in broilers [18] and turkeys [13] and increases egg weight in quails [17]. Meanwhile, betaine is a naturally existing compound in plant and animal tissue [19]. Betaine is a methyl group donor and an organic osmolyte [20]. As a methyl group donor, betaine is necessary for the transmethylation reaction. The reaction forms biologically active substances, such as carnitine, creatine, phosphatidylcholine, and epinephrine [8,20]. Betaine is also a potential osmolyte since it exerts an osmoregulatory function over epithelial cells and digestive tract microflora. Thus, it can increase the digestibility of some nutrients [8,21]. Betaine supplementation positively affects growth in chickens [22-24] and egg production in chickens, ducks, and quails [25-27].

Previous studies have addressed the effects of silicon dioxide and betaine supplementation in poultry, although the results vary [18,26]. There is also a lack of information concerning ASD and betaine supplementation in quails on follicular development and productive parameters at the onset of the laying period. It is hypothesized that the ASD and betaine supplementation could improve the quails’ growth performance and productive parameters.

This study aimed to determine the effect of ASD and betaine supplementation on quails’ growth performance during the starter period and on follicular development and productive parameters at the onset of the laying period.

## Materials and Methods

### Ethical approval

The study was approved by the Animal Ethics Committee of Sebelas Maret University under registration No. AEC-062019-022/002/2019.

### Study period and location

The study was conducted from May to July 2019 under natural tropical conditions on the experimental site belonging to Sebelas Maret University, Indonesia. During the experiment, the average ambient temperatures in the morning (06.00 h), midday (12.00 h), and evening (18.00 h) were 27.1°C, 33.6°C, and 30.1°C, respectively, and the average relative humidity levels at those times were 82.2%, 58.4%, and 66.1%, respectively.

### Quails, experimental design, and diets

The study used 1064 female quails (*C. coturnix japonica*; 7 days of age), with an average initial body weight of 23.74±1.33 g. The birds were distributed into groups receiving one of four dietary treatments in a completely randomized experimental design. Each treatment group consisted of seven replicates of 38 quails. The four treatments were as follows: A basal diet (control), a diet supplemented with 200 ppm ASD, a diet supplemented with 0.12% betaine, and a diet supplemented with a combination of 200 ppm ASD and 0.12% betaine (ASD+Betaine). The level of supplementation was based on the manufacturers’ recommendation. The dietary treatments were created by adding ASD (Silica+98.3% purity; Ceresco Nutrition, Canada) or betaine (^TNI^Betaine 96% purity; Nutreco, the Netherlands) or both. Table-1 shows the compositions of the starter basal diet and the supplemented diets.

**Table 1:** Composition and nutrient content of the basal diets.

Ingredients	Starter	Layer
Maize (%)	42.00	44.70
Rice bran (%)	13.35	7.82
Wheat bran (%)	7.25	5.00
Soybean meal (%)	26.00	25.35
Fish meal (%)	8.85	7.00
Coconut oil (%)	0.85	2.60
*DL*-methionine (%)	0.00	0.03
Dicalcium phosphate	0.00	0.60
Limestone (%)	1.10	6.30
Premix (%)^1^	0.25	0.25
NaCl (%)	0.35	0.35
Nutrient content		
Metabolizable energy (kcal/kg)	2800	2800
Crude protein (%)	22.01	20.00
Crude fat (%)	3.96	4.55
Crude fiber (%)	5.81	4.48
Crude ash (%)	6.36	7.86
Calcium (%)	1.04	3.35
Available phosphorus (%)	0.41	0.46
Lysine (%)	1.31	1.17
Methionine (%)	0.42	0.41

1 The premix supplied the following per kilogram diets: 42,000 IU Vitamin A; 7000 IU Vitamin D_3_; 28 mg Vitamin E; 7 mg Vitamin K; 7 mg Vitamin B1; 18 mg Vitamin B2; 2 mg Vitamin B6; 42 mg Vitamin B12; 88 mg Vitamin C; 21 mg calcium *D*-pantothenate; 140 mg niacin; 35 mg choline chloride; 420 mg manganese; 70 mg iron; 0.7 mg iodine; 350 mg zinc; 0.7 mg cobalt; 14 mg copper, 35 mg sanoquin (antioxidant).

The experiment followed standard management practice. The birds were raised in battery cages and had free access to both water and feed. The lighting schedule during the starting-growing period was 12 h light and 12 h dark, while during the laying period was 16 h light and 8 h dark [27].

### Growth performance

Between the ages of 7 and 42 days, the quails were given the starter diets, and, from the age of 43 days forward, they were given the layer diets. Feed consumption was measured daily, while body weights were measured weekly during the starter period until the birds were 42 days old to obtain growth performance data. The feed conversion ratio (FCR) was calculated by dividing the feed intake (FI) by weight gain [28]. The protein efficiency ratio (PER) was calculated as grams of weight gain per gram of protein intake. In contrast, the energy efficiency ratio (EER) was calculated as grams of weight gain per 100 kcal of metabolizable energy (ME) intake [28].

### Follicular characteristics and the laying performance during the onset of the laying period

Data on follicular characteristics were observed weekly from 35 to 70 days of age. Every week, two quails were chosen from each replicate and slaughtered. Their ovaries were removed immediately to measure the ovarian weights, numbers of yellow follicles, and largest follicle (F1 follicle) weights, according to Attia *et al*. [9]. During the laying period, feed consumption, egg production, and egg weight were recorded daily. Data were collected for egg production parameters from the day the quails laid their first eggs until they reached peak egg production between 13 and 15 weeks of age.

### Statistical analysis

Data were analyzed through analysis of variance according to the following model: y_ij_=μ+α_i_+ε_ij_, where, μ=the general mean; α_i_=the effect of treatment; and ε_ij_=experimental error. The mean difference (p=0.05) was analyzed using Duncan’s multiple range test, and the R Studio was applied for the statistical analyses [29].

## Results

### Growth performance

Combined supplementation with ASD+Betaine enhanced the quails’ average daily gain (ADG; 3.08 g vs. 2.76 g; p=0.01) and reduced the FCR (3.85 vs. 4.27; p=0.03) during the starting period compared with the non-supplemented diet, although the ASD and betaine single-supplement diets improved neither the ADG nor the FCR. Other growth performance parameters, including FI, PER, and EER, were not affected by these supplements at all (Table-2).

**Table 2:** Growth performance of quails fed ASD and betaine during starter period.

Treatments	FI (g/day)	ADG (g)	FCR	PER	EER
Control	11.75±0.66	2.76±0.20^b^	4.27±0.28^a^	1.07±0.07	8.39±0.53
ASD	11.63±0.65	2.80±0.13^b^	4.17±0.33^a^	1.10±0.09	8.62±0.71
Betaine	11.82±0.39	2.85±0.18^b^	4.16±0.36^a^	1.10±0.10	8.63±0.77
ASD+Betaine	11.84±0.37	3.08±0.12^a^	3.85±0.24^b^	1.18±0.07	9.30±0.59
p value	0.91	0.01	0.03	0.13	0.12

^a,b^Different superscripts in the same column indicated significant difference (p<0.05). ASD=Activated silicon dioxide; FI=Feed intake; ADG=Average daily gain; FCR=Feed conversion ratio; PER=Protein efficiency ratio; EER=Energy efficiency ratio

### Follicular characteristics

The treatments did not affect quails’ ovarian weights from 35 to 70 days of age, although a numerical increase from 56 to 70 days of age in the supplemented groups (Table-3). Furthermore, neither the follicle numbers nor the F1 follicle weights from 56 to 70 days of age were affected by the treatments (Table-4). In this study, yellow follicle numbers and F1 follicle weight was not measuredon 35, 42, and 49 days from the samples.

**Table 3:** Ovarian weight of quails fed ASD and betaine from 35 to 70 days of age (g).

Treatments	35 days	42 days	49 days	56 days	63 days	70 days
Control	0.06±0.02	0.10±0.03	0.12±0.09	1.67±0.79	3.71±1.19	4.03±1.07
ASD	0.05±0.03	0.10±0.04	0.10±0.01	1.79±0.42	4.02±1.01	5.32±0.92
Betaine	0.07±0.03	0.10±0.03	0.11±0.04	2.93±1.99	4.57±1.05	5.66±1.74
ASD+Betaine	0.07±0.02	0.11±0.06	0.10±0.05	2.02±0.61	4.59±1.17	4.93±0.47
p value	0.80	0.83	0.65	0.24	0.38	0.80

ASD=Activated silicon dioxide

**Table 4:** Follicle numbers and F1 follicle weights of quails fed ASD and betaine.

Treatments	Yellow follicle numbers* (days)			F1 follicle weight (g)* (days)		
	
	56	63	70	56	63	70
Control	2.00±1.83	3.00±0.82	3.43±0.53	1.34±0.58	1.85±0.34	2.38±0.38
ASD	3.40±0.71	3.43±0.89	3.50±0.79	1.39±1.34	1.99±0.24	2.41±0.18
Betaine	3.50±1.22	3.71±0.45	3.80±0.49	1.71±0.86	2.14±0.44	2.53±0.55
ASD+Betaine	3.20±1.30	3.71±0.89	4.00±0.49	1.18±0.52	2.35±0.71	2.40±0.09
p value	0.80	0.47	0.55	0.43	0.61	0.39

*Yellow follicle numbers and F1 follicle weight cannot be measured at 35, 42, and 49 days from the samples. ASD=Activated silicon dioxide

### Productive parameters

In this study, supplementation with ASD or betaine as a single feed additive did not affect the age at which the quails laid their first eggs, although the birds that received ASD supplementation laid their first eggs later than the other quails (p<0.04; Table-5). Similarly, the age at which the quails reached 50% egg production (EP) was not influenced by these supplements alone or in combination. However, peak production was achieved at an earlier age following ASD+Betaine supplementation than with the non-supplemented diet (15.71 weeks vs. 14.00 weeks; p=0.02).

**Table 5:** Age at some production criteria of quails fed ASD and betaine.

Treatments	First egg (week)	50% EP (week)	Peak production (week)
Control	6.76^ab^	9.00	15.71^a^
ASD	7.06^a^	9.29	14.43^b^
Betaine	6.96^ab^	8.86	14.71^b^
ASD+Betaine	6.61^b^	8.71	14.00^b^
p value	0.04	0.16	0.02

^a,b^Different superscripts in the same column indicated significant difference (p<0.05. ASD=Activated silicon dioxide; EP=Egg production

The ASD, betaine, and ASD+Betaine diets did not affect EP on 49 and 56 days of age (Table-6). However, according to data collected on 63 and 70 days of age and during peak production, these supplements enhanced egg production – particularly the betaine and ASD+Betaine diets (p<0.05). The effects of ASD supplementation were observed on 63 days of age. The greatest improvements in EP were observed with the ASD+Betaine diet, as opposed to the non-supplemented diet, on 63 (54.49% vs. 39.87%; p<0.01) and 70 days of age (69.36% vs. 53.25%; p<0.01) and during peak production (81.61% vs. 75.98%; p=0.03). The ASD, betaine, and ASD+Betaine diets did not affect egg weight on 49 days of age (Table-7). However, on 56, 63, and 70 days of age and during peak production, these supplements increased egg weight (p<0.05).

**Table 6:** Egg production at the onset of laying period of quails fed ASD and betaine (%)

Treatments	49 days	56 days	63 days	70 days	Peak production
Control	2.14±1.93	22.09±3.30	39.87±2.45^d^	53.25±4.62^c^	75.98±5.53^b^
ASD	2.54±1.40	21.74±4.26	45.67±2.47^c^	56.12±1.75^c^	75.26±4.99^b^
Betaine	3.48±2.49	24.74±3.08	49.48±3.59^b^	62.75±4.78^b^	78.73±4.23^a^
ASD+Betaine	3.14±2.23	23.62±4.69	54.49±2.90^a^	69.36±4.26^a^	81.61±2.59^a^
p value	0.85	0.07	<0.01	<0.01	0.03

^a,b,c,d^Different superscripts in the same column indicated significant difference (p<0.05). ASD=Activated silicon dioxide

**Table 7:** Egg weight at the onset of laying period of quails fed ASD and betaine (g).

Treatments	49 days	56 days	63 days	70 days	Peak production
Control	8.75±0.88	8.90±0.64^b^	9.00±0.41^b^	9.00±0.49^b^	9.30±0.27^b^
ASD	8.98±0.25	9.40±0.48^a^	9.50±0.30^a^	9.85±0.67^a^	9.92±0.19^a^
Betaine	8.91±0.54	9.54±0.35^a^	9.65±0.27^a^	9.82±0.64^a^	9.97±0.36^a^
ASD+Betaine	8.60±0.38	9.50±0.34^a^	9.73±0.80^a^	9.91±0.37^a^	9.92±0.29^a^
p value	0.7	0.04	<0.01	<0.01	<0.01

^a,b^Different superscripts in the same column indicated significant difference (p<0.05). ASD=Activated silicon dioxide

## Discussion

### Growth performance

In this study, neither ASD nor betaine improved growth performance in quails when supplemented as single feed additives. However, a synergistic effect between the supplements was observed in the ASD+Betaine diet, as the quails’ ADG and FCR improved. Previous observations have indicated that adding betaine to quails’ diets containing 20% crude protein (CP) increases weight gain [30], a finding that differs from the results of this study. However, the starter diet in this research contained 22% CP, which may have affected the betaine efficacy [31]. Betaine donates its methyl groups to form methylated compounds, including methionine, contributing to protein metabolism [20]. The efficacy of betaine as methyl group donor decreases with increasing protein content in the diet [32]. Other observations have indicated that betaine improves weight gain and feed conversion in broiler chickens [24,32] and meat-type ducks [33], though still, other studies have not found any effects of betaine on growth performance in broiler chickens [34]. Tran *et al*. [13] have observed that silicon dioxide supplementation improves weight gain and feed conversion in turkey. Dietary silicate minerals have also enhanced the EER and PER in broilers [15]. However, studies using broilers [14] and turkey [35] have shown no growth performance improvement following ASD supplementation. Recent research indicates that silicon dioxide supplementation did not affect quails’ growth until they reach the age of 7 weeks, but its effect increases at weeks 8 and 9 [36].

Both ASD and betaine can enhance nutrient absorption in the intestine [14,17,27], although numerical increases in ADG and FCR due to these supplements when included as single feed additives were not significant. When applied together, ASD and betaine facilitate higher nutrient availability and absorption, leading to improved ADG and FCR. Dietary silicate minerals reduce the gastrointestinal passage rate that nutrients are exposed to digestion longer [15], while betaine improves the morphology of intestinal cells, providing a more intact area surface for nutrient absorption [8]. Silicon dioxide and betaine help nutrient digestibility by improving the intestinal lumen’s environment and decreasing osmolality [8,15,17,37]. In all cases, the efficacy of feed additive supplementation is affected by several factors, including the species and type of poultry, the environmental conditions, and the nutrient content of the diet [23].

### Follicular characteristics

The ovarian weight gradually increased as the quails grew older. Ovarian size is influenced by the number and weight of those follicles. In this quail species, the ovaries are grape-shaped organs responsible for egg follicle growth and maturation [38]. In mature quails, the ovarian weight ranged between 3.5 and 6.5 g [39]. Follicles had not developed in the ovaries between 35 and 49 days of age but had begun developing by 56 days, as evidenced by the follicular weight measured on 56-70 days. Previous findings have shown that betaine stimulates the anterior pituitary gland to increase the secretion of follicle-stimulating and luteinizing hormones. These hormones stimulate follicular growth and increase EP [40]. However, the non-significant effect of ASD+Betaine supplementation on ovarian weight in this research could have been caused by these supplements having no effect on follicle development during the starting or growing periods and at the onset of the laying period. The synergistic effect of ASD and betaine in increasing the nutrient absorption during these periods was expressed in growth performance improvement rather than improvement in follicular growth and productive performance. This finding was per the improvement in ADG and FCR of the ASD+Betaine group. Previous research indicates that betaine supplementation did not affect EP in quails at the onset of the laying period, a result likely due to a high standard deviation [41]. Similarly, a study in quails indicated that betaine did not affect EP during 42-63 days, but the effect of betaine is more apparent after 70 days of age [42]. No study has been previously conducted concerning the effects of silicon dioxide supplementation on follicular characteristics. However, observations in laying hens showed that dietary inclusion of silicon [43] or nano-silicon dioxide [17] did not improve EP, indicating that silicon did not affect follicle development.

The single left ovary in *C. coturnix japonica* contains a hierarchy of growing follicles, gradually progressing toward maturity [44]. Large pre-ovulatory follicles are selected from the pool of small follicles and enter a rapid growth phase, while the vast majority become atretic [38,40]. These developing follicles are categorized by size and color: Large, white or small, and yellow follicles [40]. Follicle development to maturation in *Coturnix* quails is rapid, which occurs about 6 days [45]. This research observed the enhancement of F1 follicle numbers and weights when the quails were 56-70 days. Thus, the observed follicles were still immature, and EP was increasing toward peak production. Follicular maturation occurs when an ovum ceases to develop rapidly. Mature follicles can only be observed during a short time since the rest period between follicle maturation and oviposition is 0.1 days [40,45].

Follicle weight and numbers increase as body age increases [45]. In addition, at the onset of the laying period, the bodyweight of quails is still increasing, influencing ovary and follicle size. Quails with body weights of 120-130 g have an F1 follicle weight of 2.31 g, while quails with body weights of 150-160 g have F1 follicle weights of 3.34 g [46]. In most birds, ovaries show five follicle sizes (largest to smallest: F1, F2, F3, F4, and F5); small, yellow follicles, and numerous white follicles [38,47]. Ovarian follicular activities and hierarchies may be attributed to genetics, as well as to nutritional and management conditions [40,47].

### Productive parameters

Supporting the finding in productive parameters, Ratriyanto [42] did not observe any influence on age at the start of the laying period or on 50% EP when betaine was added to quails’ diets containing 18.7% protein. In that study [42], the first egg was laid when the quails were 6 weeks of age, and 50% EP was achieved after 8 weeks of age. These results were in the normal range for quails, which reach sexual maturity at about 6-7 weeks of age and reach a peak EP at approximately 15 weeks of age [1,6]. As with the follicular characteristics above, there was no literature examining silicon dioxide supplementation on age at various production parameters in quails.

The ASD, betaine, and ASD+Betaine diets did not affect EP on 49 and 56 days of age (Table-6). However, according to data collected on 63 and 70 days of age and during peak production, these supplements enhanced egg production – particularly the betaine and ASD+Betaine diets (p<0.05). The effects of ASD supplementation were observed only on 63 days of age. The highest improvements in EP were observed with the ASD+Betaine diet, as opposed to the non-supplemented diet, on 63 (54.49% vs. 39.87%; p<0.01) and 70 days of age (69.36% vs. 53.25%; p<0.01) and during peak production (81.61% vs. 75.98%; p=0.03). The greatest improvement observed in ASD+Betaine group could be attributed to the synergistic effect of ASD and betaine in improving nutrient absorption leading to higher nutrient availability for EP. As explained previously, ASD and betaine can increase nutrient absorption in the intestine [14,17,27]. EP on 49 and 56 days of age was still relatively low since the quails were in the early stages of the production period, and production was not yet stable [7]. The low rate of follicle recruitment could cause low EP at the onset of the laying period into the yellow follicle hierarchy [39]. The previous studies indicate that the effects of betaine supplementation are observable at 10 weeks of age [41,42].

The productive performance improvement following the ASD and betaine diets may be attributable to increased nutrient availability for EP. Betaine improves nutrient digestibility [9,31], which further enhances productive traits, such as EP, egg weight, and feed conversion, in laying poultry [9,26], ducks [25], and quails [27,31]. Betaine supplementation, at 0.10%, has increased the yolk weight of chicken eggs [9]. Another study shows that betaine supplementation in quails’ diets, containing 2800 kcal/kg ME and 17% protein, enhanced egg weight by 3.82% [31]. Betaine also increases the levels of estrogen and progesterone in the blood. These hormones regulate ovulation and oviductal development [48]. ASD acts as a catalyst, accelerating biochemical changes in the digestive tract, which improves nutrient absorption [37]. Safaeikatouli *et al*. [15] illustrated that silicate minerals make temporary links with nutrients, decrease gastrointestinal transmission rates, and allow nutrients to digest further, thus increasing nutrient absorption. A previous study demonstrates that nano-silicon dioxide supplementation in quails’ diets does not affect EP but increases egg weight during 16-20 weeks of age [17].

## Conclusion

Only ASD+Betaine supplementation improved quails’ growth performance during the starter period. However, ASD and betaine, either as single feed additives or combined, did not affect ovarian and follicular characteristics at the onset of the laying period. ASD and betaine also improved quails’ productive parameters, as indicated by enhanced EP and weight; the highest magnitude was achieved through the ASD+Betaine diet. Thus, combined ASD and betaine could be beneficially applied to quails’ diets during the starter period and at the onset of the laying period.

## Authors’ Contributions

AR: Conception and design, conducted the experiment, data acquisition, data analysis and interpretation, manuscript drafting, and revision. SP: Conception and design, manuscript drafting, and revision. NW: Data analysis and interpretation and manuscript drafting. All authors read and approved the final manuscript.
